# Critical assessment of transformer-based AI models for German clinical notes

**DOI:** 10.1093/jamiaopen/ooac087

**Published:** 2022-11-15

**Authors:** Manuel Lentzen, Sumit Madan, Vanessa Lage-Rupprecht, Lisa Kühnel, Juliane Fluck, Marc Jacobs, Mirja Mittermaier, Martin Witzenrath, Peter Brunecker, Martin Hofmann-Apitius, Joachim Weber, Holger Fröhlich

**Affiliations:** Department of Bioinformatics, Fraunhofer Institute for Algorithms and Scientific Computing (SCAI), Schloss Birlinghoven, Sankt Augustin, Germany; Bonn-Aachen International Center for Information Technology (B-IT), University of Bonn, Bonn, Germany; Department of Bioinformatics, Fraunhofer Institute for Algorithms and Scientific Computing (SCAI), Schloss Birlinghoven, Sankt Augustin, Germany; Institute of Computer Science, University of Bonn, Bonn, Germany; Department of Bioinformatics, Fraunhofer Institute for Algorithms and Scientific Computing (SCAI), Schloss Birlinghoven, Sankt Augustin, Germany; Knowledge Management, ZB MED – Information Centre for Life Sciences, Cologne, Germany; Graduate School DILS, Bielefeld Institute for Bioinformatics Infrastructure (BIBI), Faculty of Technology, Bielefeld University, Bielefeld, Germany; Knowledge Management, ZB MED – Information Centre for Life Sciences, Cologne, Germany; The Agricultural Faculty, University of Bonn, Bonn, Germany; Department of Bioinformatics, Fraunhofer Institute for Algorithms and Scientific Computing (SCAI), Schloss Birlinghoven, Sankt Augustin, Germany; Department of Infectious Diseases and Respiratory Medicine, Charité – Universitätsmedizin Berlin, Corporate Member of Freie Universität Berlin, Humboldt-Universität zu Berlin, Berlin, Germany; Berlin Institute of Health (BIH) at Charité – Universitätsmedizin Berlin, Berlin, Germany; Department of Infectious Diseases and Respiratory Medicine, Charité – Universitätsmedizin Berlin, Corporate Member of Freie Universität Berlin, Humboldt-Universität zu Berlin, Berlin, Germany; German Center for Lung Research (DZL), Partner Site Charité, Berlin, Germany; Berlin Institute of Health at Charité – Universitätsmedizin Berlin, Core Facility Research IT, Berlin, Germany; Department of Bioinformatics, Fraunhofer Institute for Algorithms and Scientific Computing (SCAI), Schloss Birlinghoven, Sankt Augustin, Germany; Bonn-Aachen International Center for Information Technology (B-IT), University of Bonn, Bonn, Germany; Berlin Institute of Health (BIH) at Charité – Universitätsmedizin Berlin, Berlin, Germany; Charité – Universitätsmedizin Berlin, Center for Stroke Research Berlin, Corporate Member of Freie Universität Berlin and Humboldt-Universität zu Berlin, Berlin, Germany; Department of Neurology, Charité – Universitätsmedizin Berlin, Corporate Member of Freie Universität Berlin and Humboldt-Universität zu Berlin, Berlin, Germany; Department of Bioinformatics, Fraunhofer Institute for Algorithms and Scientific Computing (SCAI), Schloss Birlinghoven, Sankt Augustin, Germany; Bonn-Aachen International Center for Information Technology (B-IT), University of Bonn, Bonn, Germany

**Keywords:** clinical concept extraction, natural language processing, transformer-based models

## Abstract

**Objective:**

Healthcare data such as clinical notes are primarily recorded in an unstructured manner. If adequately translated into structured data, they can be utilized for health economics and set the groundwork for better individualized patient care. To structure clinical notes, deep-learning methods, particularly transformer-based models like *Bidirectional Encoder Representations from Transformers (BERT)*, have recently received much attention. Currently, biomedical applications are primarily focused on the English language. While general-purpose German-language models such as GermanBERT and GottBERT have been published, adaptations for biomedical data are unavailable. This study evaluated the suitability of existing and novel transformer-based models for the German biomedical and clinical domain.

**Materials and Methods:**

We used 8 transformer-based models and pre-trained 3 new models on a newly generated biomedical corpus, and systematically compared them with each other. We annotated a new dataset of clinical notes and used it with 4 other corpora (BRONCO150, CLEF eHealth 2019 Task 1, GGPONC, and JSynCC) to perform named entity recognition (NER) and document classification tasks.

**Results:**

General-purpose language models can be used effectively for biomedical and clinical natural language processing (NLP) tasks, still, our newly trained BioGottBERT model outperformed GottBERT on both clinical NER tasks. However, training new biomedical models from scratch proved ineffective.

**Discussion:**

The domain-adaptation strategy’s potential is currently limited due to a lack of pre-training data. Since general-purpose language models are only marginally inferior to domain-specific models, both options are suitable for developing German-language biomedical applications.

**Conclusion:**

General-purpose language models perform remarkably well on biomedical and clinical NLP tasks. If larger corpora become available in the future, domain-adapting these models may improve performances.

## INTRODUCTION

In many countries, a considerable portion of clinical routine information is still not gathered in a structured format. While structured data are commonly utilized for health economics and registries, it often lacks specific information, such as descriptions of adverse drug events, disease severity, family history, or behavioral and environmental health determinants. Such information is predominantly documented in clinical free-text form, which makes up to 40% of the data generated in current hospital systems.^1^ The great potential of information documented in narrative text to support translational research and the implementation of clinical applications was recognized early,^2–4^ but exploiting that potential still poses a challenge. Extracting clinical information through natural language processing (NLP) methods could structure that information to support downstream clinical applications such as deep phenotyping, better-individualized clinical decision-making, and automated coding for health economic purposes.

Nowadays, the development of NLP systems for information extraction in English is already quite advanced. Systems such as MedLEE,^2^^,^^5^ MetaMap,^6^ cTAKES,^7^ and CLAMP^8^ have been developed and deployed in the past to extract information from clinical narrative texts. Furthermore, open competitions such as *Informatics for Integrating Biology and the Bedside* (i2b2),^9^*National NLP Clinical Challenges* (n2c2),^10^^,^^11^ and *CLEF eHealth*^12^ encourage sharing of data and models and are further driving developments in this area. The systems developed so far include rule-based, machine-learning-based, and hybrid models. While rule-based approaches were indispensable in the early stages, today’s research often focuses on machine-learning methods. In particular, deep-learning networks, such as recurrent neural networks (RNNs) or convolutional neural networks, have been used extensively in recent years^13^ as they can achieve higher performances if sufficient amounts of training data exist. Compared to traditional machine-learning methods, deep neural networks usually employ methods such as Word2Vec,^14^^,^^15^ GloVe,^16^ or FastText^17^ to represent words as vectors. These methods model language by learning relationships between words – so-called word embeddings – from a large textual corpus. Using the word embeddings as features replaces the manual feature engineering required by traditional methods. Following the idea of word vector representation, research continued and led to the development of another group of deep neural networks – transformer-based models. The Transformer, published by Vaswani et al. in 2017,^18^ was initially designed for neural machine translation and addressed two shortcomings of RNNs: missing parallelization and long-range dependencies. It relies heavily on the self-attention mechanism, which weighs each part of the input differentially. Since it works without recurrence, it is parallelizable and computationally more efficient than the RNN counterpart. In 2019, Devlin et al. used parts of the original architecture to develop *Bidirectional Encoder Representations from Transformers* (BERT) and achieved state-of-the-art results in numerous NLP tasks.^19^ As with other transformer-based models, it is trained in 2 stages: First, it is pre-trained using large amounts of unlabeled data by applying novel training objectives such as masked language modeling (MLM) and next-sentence prediction. In the second stage, the model is fine-tuned for specific NLP tasks with labeled data. Since the publication of BERT, numerous variants of the model have been presented. While approaches such as RoBERTa^20^ and ELECTRA^21^ tackled potential limitations and shortcomings of the model architecture and training procedure, other variants such as BioBERT^22^ and ClinicalBERT^23^^,^^24^ were developed to achieve domain specificity.

In the German-speaking world, developments lag far behind and are often driven only by commercial software or local applications.^25^ Strict data protection laws hinder data sharing, and thus clinics typically only allow for the use of data internally. These factors inhibit the sharing of datasets and models, as well as the hosting of open challenges with German datasets.^25^^,^^26^ Nevertheless, there have been promising approaches in recent years: With JSynCC^27^ and GGPONC,^28^ 2 datasets have been published that contain texts with biomedical language but are not affected by data protection issues. Recently, the first corpus containing de-identified discharge letters, called BRONCO150,^29^ was published. Furthermore, the CLEF eHealth challenge provided a dataset of non-technical summaries of animal studies in 2019. Sänger et al. used the multilingual BERT version (mBERT) to classify these summaries and showed that mBERT significantly outperformed a baseline Support Vector Machine model.^30^ Later, Bressem et al. trained domain-specific BERT models using 3.8 million radiographic reports and evaluated them in a classification task with promising results. Similarly, Richter-Pechanski et al. pre-trained BERT models on 200 000 discharge letters and fine-tuned them for a clinical concept extraction task. General-purpose language models (GPLMs) have already performed excellently in all of these cases. However, none of these studies systematically compared already published models such as GottBERT or GELECTRA, but rather focused on mBERT or GermanBERT. Furthermore, none of the pre-trained clinical models are publicly available yet.

In our work, we developed 3 new biomedical domain-specific GPLMs and evaluated their performance on 5 clinical NLP tasks in comparison to 8 GPLMs. For this purpose, we first assembled a dataset of unlabeled biomedical texts and trained our models. We then annotated clinical entities in 50 discharge letters to generate a new dataset called ChaDL (**Cha**rité **D**ischarge **L**etters), which we used with BRONCO150, the CLEF eHealth dataset from 2019, GGPONC, and JSynCC to fine-tune and evaluate models. To our knowledge, this is the first comprehensive comparison of German-language transformer models for clinical NLP applications.

## MATERIALS AND METHODS

### General overview

**Figure 1.**
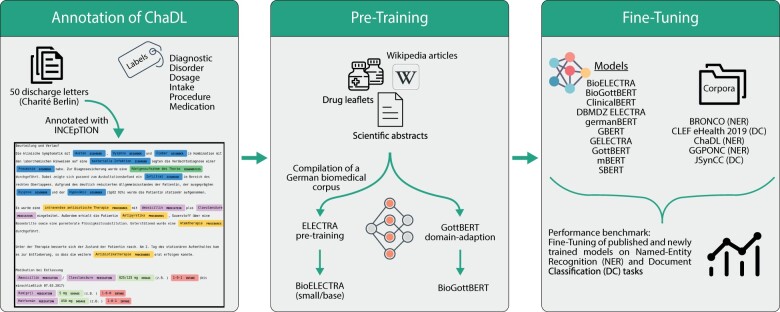
Study overview. First, a set of 50 discharge letters was annotated with medical entities. Second, biomedical transformer models were pre-trained on a newly assembled biomedical corpus by either training it from scratch or through domain adaption of an existing model. Third, the pre-trained models were compared to 8 published models on 5 fine-tuning tasks.

The work described in this article consisted of 3 phases (Figure 1):

1. Annotation of ChaDL: We manually annotated 50 de-identified discharge letters from the Charité – Universitätsmedizin Berlin with respect to the entities diagnosis, disorder, dosage, intake, medication, and procedure.
2. Pre-Training: Subsequently, we pre-trained several transformer models on German-language scientific abstracts, drug leaflets, and medicine-related Wikipedia articles.
3. Fine-Tuning: Finally, we performed fine-tuning and evaluation of eleven models for named entity recognition (NER) and document classification based on 5 corpora, including ChaDL.

In the following, we describe our approach in more detail.

### Datasets

We used 6 different datasets for pre-training and fine-tuning of transformer models. The corpora we compiled for pre-training consisted of German medical articles from Wikipedia, drug leaflets from the AMIce database (https://www.dimdi.de/dynamic/de/arzneimittel/arzneimittel-recherchieren/amis/), and scientific abstracts from the LIVIVO search engine.^33^ For the latter, we only used abstracts from databases with biological or medical relevance. All elements such as lists, tables, and equations that can confuse text mining systems were removed from the documents. As shown in Supplementary Table A.1, the corpus contains approximately 0.8 GB of textual data and 66 million tokens.

For fine-tuning, we used 4 publicly available datasets, namely BRONCO150,^29^ the CLEF eHealth 2019 dataset,^34^ GGPONC,^28^ and JSynCC,^27^ and a newly created dataset of clinical discharge letters called ChaDL that originated from Charité – Universitätsmedizin Berlin.

JSynCC is the first publicly available dataset with documents in the German clinical language. It contains 867 documents extracted from 10 medical textbooks (see Table 1). Since each document is assigned to one or more specialized medical fields, this dataset is suited for a multi-label document classification task. Nonetheless, the class distribution is highly imbalanced, and most labels are only represented a few times (see Supplementary Figure A.5). Since a model can neither be adequately trained nor evaluated if classes are this scarcely represented, we generated 2 subsets of JSynCC in which we excluded classes whose frequency does not exceed a specified threshold. Version A represents the extreme case in which only a few samples are available to train a model: We kept all document labels that occurred at least 5 times, thereby reducing the number of documents from 867 to 849. For version B, which is closer to a real-world scenario with more samples available for training, we limited labels to those that occur at least 50 times, thereby reducing the total documents from 867 to 494. The main article shows the results of our experiments with version B. A detailed description of version A and the respective results are available in Supplementary Appendix C.2.

**Table 1.** The overview of the datasets provides details about the number of documents, sentences, and tokens as well as the number of instances for each class

	BRONCO150	ChaDL	GGPONC	JSynCC (version B)	CLEF
*Textual elements*					
Documents/segments	150	50 (225^a^)	4153	494	8792
Sentences	8976	2527	29 528	20 971	200 989
Tokens	70 572	31 920	664 029	275 700	3 332 420
*Entities*					
Anatomical structure	–	–	3825	–	–
Chemical drugs	–	–	8335	–	–
Devices	–	–	1519	–	–
Diagnostics	–	349	–	–	–
Diagnosis	3473	–	–	–	–
Disorder	–	1901	18 721	–	–
Dosage	–	301	–	–	–
Intake	–	315	–	–	–
Living beings	–	–	10 363	–	–
Medication	1233	579	–	–	–
Physiology	–	–	4848	–	–
Procedures	–	515	23 741	–	–
Treatment	2320	–	–	–	–
TNM	–	–	1081	–	–
*Document classes*					
Accident surgery	–	–	–	266	–
Emergency medicine	–	–	–	107	–
Orthopedics	–	–	–	282	–
Traumatology	–	–	–	50	–

*Note:* Classes that are not present in one of the datasets are denoted with “–”. For the CLEF eHealth 2019 dataset, we only report the number of documents, sentences and tokens, as more than 200 possible labels exist.

a Number of sections which were extracted from the discharge letters.

As part of 2019s CLEF eHealth challenge, a dataset comprising 8793 German NTPs of animal experiments was made available. The documents have been manually annotated by experts; each has received zero or more ICD-10 codes as document-level label. Like JSynCC, we used it for a multi-label document classification task.

GGPONC contains 8414 text segments that have been extracted from 25 oncology clinical practice guidelines and hence is one of the largest corpora of German medical texts. Borchert et al. automatically annotated the corpus with 7 UMLS terms and screened for TNM expressions and gene names. Afterward, 4 annotators manually curated a subset of 4153 text segments to generate a gold standard. In this study, we used only the 4153 manually curated text segments for our experiments.

As the first freely available corpus of de-identified clinical notes, the recently published Berlin-Tübingen Oncology corpus (BRONCO150) contains shuffled sentences from 150 German oncological discharge summaries. Nine annotators (medical experts and students) annotated the documents using the labels diagnosis, treatments, medication, and other attributes.

Our newly created dataset ChaDL consists of 50 de-identified discharge letters from the neurological department of the Charité – Universitätsmedizin Berlin, collected as part of studies in which informed consent was given to extract data from the hospital information system. These discharge letters contain various sections from which we focused on *anamnesis, diagnoses, medication, and epicrisis*. We used the annotation tool INCEpTION^35^ to manually annotate the mentions for diagnostic, disorder, dosage, intake, medication, and procedure entity classes (see Supplementary Material Section A.2 for details of the annotation process). These entity classes were chosen to capture detailed information about patients’ examination, health condition, and treatment. The majority of the discharge letters were annotated by only 1 annotator; however, 20% were annotated by a second expert to determine the quality of manual annotation by calculating the inter-annotator agreement score Krippendorff’s alpha. On average, we achieved a score of 0.76 ± 0.11, indicating a relatively high agreement between the 2 annotators.

### Published transformer models

We focused our experiments on the 3 transformer-based model architectures BERT, ELECTRA, and RoBERTa.

**BERT** ^19^ is a bidirectional transformer-based encoder model, which is pre-trained on large amounts of unlabeled data using MLM and next sentence prediction (NSP) jointly as training objectives. During MLM, some input tokens are randomly masked and the objective is to predict the original tokens based only on their context. The NSP task is to determine if 2 sentences are consecutive or not.

**RoBERTa** ^20^ is an optimized version of BERT. It is built on the same architecture as BERT but abandons the NSP objective and only uses masked-language modeling for pre-training. Unlike BERT, however, the data are not masked statically during preprocessing but dynamically during each epoch. In addition, some hyperparameters such as the batch size and the tokenizer have been changed.

**ELECTRA** ^21^ uses the same architecture as BERT but differs in its pre-training procedure. While BERT aims for MLM and NSP, ELECTRA uses a method called replaced token detection (RTD). Two separate models are used for this purpose: a generator and a discriminator. The generator is trained by MLM, and its output is then used as input for the discriminator. The discriminator has to predict whether a token has been replaced or whether it is the original input. After pre-training, only the discriminator is used.

Table 2 lists the models we used in this study and provides information on the data used for pre-training. All German language models were trained on general language corpora consisting of Wikipedia articles, books, news articles, or vast amounts of crawled textual data.

**Table 2.** Overview of the published models

Model name	Data	Corpus size (GB)
ClinicalBERT^24^	MIMIC-III^36^	–
DBMDZ ELECTRA model^37^	Europeana newspapers^a^	51.0
GermanBERT^b^	German Wikipedia, OpenLegalData,^38^ and news articles	12.0
GBERT^39^	German Wikipedia, OpenLegalData, OPUS,^40^ and OSCAR^41^	163.4
GELECTRA^39^	”	”
GottBERT (RoBERTa)^42^	OSCAR^41^	145.0
multilingual BERT (mBERT)^c^	Wikipedia of 100+ languages	–
Sentence-BERT (SBERT)^d^	Paraphrase dataset of 50+ languages	–

*Notes*: Names and information about the data used for pre-training for each of the 8 publicly available models. Unabridged dataset names: Open Super-large Crawled Aggregated coRpus (OSCAR), Open Parallel corpUS (OPUS), and Medical Information Mart for Intensive Care (MIMIC-III).

“–” indicates missing information about the dataset size.

a http://www.europeana-newspapers.eu/. Accessed March 2, 2022.

b https://deepset.ai/german-bert. Accessed March 2, 2022.

c https://github.com/google-research/bert. Accessed March 2, 2022.

d https://huggingface.co/T-Systems-onsite/german-roberta-sentence-transformer-v2. Accessed March 2, 2022.

### Training and assessment of language models for the German clinical domain

#### Pre-training

We followed 2 strategies to pre-train transformer models specific to the German biomedical domain. First, we used an existing RoBERTa-based model, named GottBERT, for domain adaption, and second, we trained 2 newly initialized ELECTRA-based models from scratch.

For the domain-adapted GottBERT model, we loaded the pre-trained model and trained it on our biomedical corpus with static masked-language modeling and linear learning rate scheduling. A detailed list of used hyperparameters can be found in Supplementary Table B.1. We denote this model as BioGottBERT.

For the ELECTRA models, we used our biomedical corpus to generate a new vocabulary for the WordPiece tokenizer.^43^ Then, we initialized 2 new ELECTRA models in both the *small* and *base* configurations and subsequently trained both with the hyperparameters specified in Supplementary Table B.1. We refer to these 2 models as BioELECTRA-small and BioELECTRA-base, respectively.

#### Performance assessment

We assessed the performance of the 8 published and the 3 pre-trained transformer-based models on 2 types of downstream tasks, document classification and NER.

The JSynCC and the CLEF eHealth datasets (see Table 1) were used to evaluate the models for multi-label document classification tasks. For the transformer-based models, the documents were split into one or more sequences of 512 tokens. If multiple instances existed per document, max-pooling was applied to the logits before loss calculation and final classification.

For the NER task, BRONCO150 (we used the same 5 outer folds as the authors to evaluate the model performances), GGPONC, and ChaDL (see Table 1) were used for the performance assessment. The data were prepared according to the BILOU tagging scheme, and the performance was assessed at the entity level.

In all fine-tuning studies, we fine-tuned the transformer-based models and compared their performances to a baseline. In the case of the CLEF dataset, we compare performance to the best result the challenge organizers provided. In all other experiments, we trained a bidirectional LSTM network with a Conditional Random Field (Bi-LSTM-CRF). When we trained the models for the CLEF dataset, we used the train, validation, and test splits from the original tasks. In all other cases, we performed 5-fold nested cross-validation to assess the performance of the models. We used the *Optuna* hyperparameter optimization framework^44^ to optimize hyperparameters such as the batch size, learning rate, and weight decay (see Supplementary Table B.2 for details) by maximizing the micro *F*_1_-score. We trained for a maximum of 50 (BRONCO150, ChaDL, GGPONC, JSynCC) or 80 (CLEF eHealth 2019) epochs but used an early stopping procedure to stop after 15 epochs if performance did not improve ($\Delta F_{1}<0.01$); the best model was used for evaluation in the end.

### Implementation

The tokenizers and transformers libraries developed by the HuggingFace team were used for pre-training and fine-tuning experiments of the transformer-based models. For the training of the Bi-LSTM-CRF model, we used the *flair* framework with GloVe and *flair* embeddings.^45–47^ For pre-training, we utilized up to 4 NVIDIA V100 or A100 GPUs. In all other cases, single NVIDIA V100 or A100 GPUs were used.

We used several libraries to calculate metrics: The *kAlpha* (https://github.com/emerging-welfare/kAlpha, accessed on November 24, 2021) implementation was used to calculate Krippendorff’s Alpha for the inter-annotator agreement. The metrics for the multi-label document classification tasks were calculated with the *classification_report* function from *scikit-learn* (version 0.23.2),^48^ and the metrics for the NER tasks were calculated with *classification_report* function from the *seqeval* library (version 1.2.2).^49^

## RESULTS

In this study, we show the assessment results of general-purpose and domain-specific language models for the German clinical domain. We begin by presenting the pre-training results of the 3 models. Then, we highlight the fine-tuning performance of these 3 newly pre-trained and 8 already-published models on the 5 fine-tuning tasks.

### Pre-training performance

**Figure 2.**
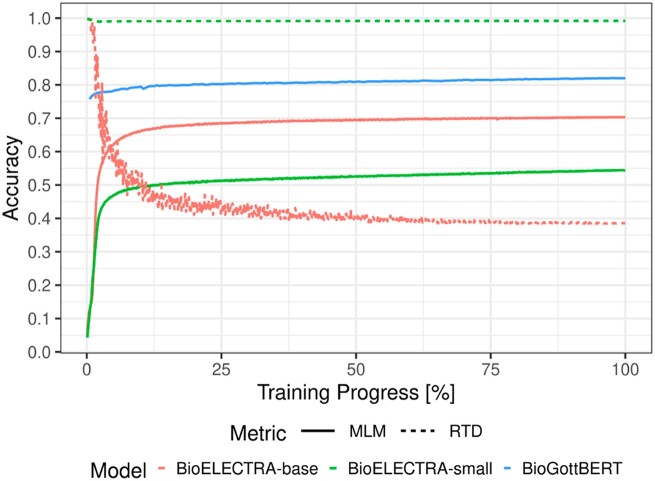
Pre-training accuracy. Overview of the pre-training performances for the BioELECTRA-base, BioELECTRA-small, and BioGottBERT models. The masked-language modeling (MLM) accuracy was calculated for all model types, while the replaced-token detection (RTD) could only be calculated for the BioELECTRA-base and BioELECTRA-small models.

Figure 2 shows the pre-training metrics of the 3 new models. In the case of BioGottBERT, where we followed a transfer-learning approach and initialized it with the GottBERT parameters, the MLM accuracy increased from 75 to 82.0%. Unfortunately, a direct comparison of the BioGottBERT metrics and those of the BioELECTRA-small and BioELECTRA-base models is problematic since different training objectives were followed. For these 2 models, there are 2 measures, namely MLM and RTD accuracy. In both cases, the generators’ MLM accuracy starts at 0% and moves, after an initial sharp increase, to 54%, and 70% for the small and base models. On the other hand, the discriminators’ RTD accuracy starts at close to 100% and deteriorates to 39% for the base model, whereas in the small model, it ends at 99%. A subsequent examination of the training’s environmental impact revealed that the training of BioELECTRA-small and BioGottBERT required comparable amounts of energy; however, BioELECTRA-base required approximately 4 times more (see Supplementary Appendix B.2).

### Fine-tuning performance

Table 3 depicts the results of the document classification tasks on the CLEF eHealth 2019 and JSynCC (see Supplementary Table C.4 for the results of subset version A) datasets. For JSynCC, all models, including the Bi-LSTM-CRF model, achieved very high *F*_1_-scores ranging from 89.0 to 92.7%. The greatest *F*_1_-scores were obtained by GBERT, mBERT, and GermanBERT, with no significant difference between them. When applied to the CLEF eHealth dataset, the differences between the results increased substantially. Our fine-tuned variant was slightly inferior to Sänger et al’s mBERT model ($\Delta F_{1}=-1.2$); however, GottBERT and GBERT reached a comparable result. In both cases, our pre-trained BioELECTRA and BioGottBERT models were outperformed by the top-performing GBERT model.

**Table 3.** Model performances of the document classification task on the CLEF eHealth 2019 and JSynCC datasets

	CLEF			JSynCC (version B)		
*Model*	F1	Pre.	Rec.	F1	Pre.	Rec.
BioELECTRA-base	74.3	78.2	70.9	91.2 (1.6)	87.4 (4.5)	95.6 (2.1)
BioELECTRA-small	76.9	79.7	74.2	90.3 (1.7)	84.2 (3.6)	**97.5 (1.7)**
BioGottBERT	77.2	81.0	73.7	89.0 (1.9)	84.3 (3.4)	94.7 (6.2)
ClinicalBERT	–	–	–	–	–	–
DBMDZ ELECTRA	76.4	79.6	73.6	91.4 (2.0)	86.8 (3.2)	96.7 (2.6)
GBERT	**80.3**	**84.6**	76.4	**92.7 (2.3)**	**90.4 (4.4)**	95.4 (3.7)
GELECTRA	74.4	75.7	73.2	91.4 (1.3)	86.5 (2.2)	96.9 (1.9)
GottBERT	79.5	81.7	**77.4**	89.5 (2.6)	88.2 (2.5)	91.3 (6.8)
GermanBERT	76.7	79.2	74.3	91.8 (2.0)	89.9 (4.2)	94.0 (3.5)
mBERT	78.8	83.9	74.2	91.9 (1.7)	88.2 (3.9)	96.2 (2.6)
sBERT	73.8	77.1	70.8	90.3 (2.1)	86.2 (3.3)	95.1 (4.7)
Bi-LSTM-CRF	–	–	–	91.3 (3.6)	89.9 (3.8)	93.5 (9.3)
Sänger et al. (mBERT)	**80**	83	**77**	–	–	–

*Notes*: This table shows the micro-averaged scores for each model. Since nested cross-validation was performed for JSynCC, the mean and standard deviation are reported. The best-performing models are highlighted in bold.

The results of the 3 NER tasks, in which various medical entities are detected in BRONCO150, ChaDL, and GGPONC corpora, are summarized in Table 4. In contrast to the GGPONC dataset, the model performances vary considerably on BRONCO150 and ChaDL datasets.

**Table 4.** Overview on scores achieved for NER task on 3 datasets

	BRONCO150			ChaDL			GGPONC		
*Model*	F1	Pre.	Rec.	F1	Pre.	Rec.	F1	Pre.	Rec.
BioELECTRA-base	64.1 (6.0)	55.6 (9.2)	76.8 (2.5)	55.3 (10.5)	47.1 (13.8)	70.4 (3.6)	65.1 (7.3)	54.8 (10.4)	81.6 (0.4)
BioELECTRA-small	46.7 (10.3)	34.5 (10.4)	74.8 (8.1)	61.1 (6.5)	52.9 (10.4)	74.2 (3.2)	82.3 (0.4)	81.9 (0.7)	82.6 (0.7)
BioGottBERT	**83.2 (1.6)**	**83.5 (1.2)**	**82.9 (2.1)**	**80.4 (1.1)**	**81.3 (1.5)**	79.6 (2.2)	83.8 (0.4)	83.7 (1.2)	84.0 (0.9)
ClinicalBERT	–	–	–	44.4 (6.0)	42.4 (7.3)	47.4 (6.5)	–	–	–
DBMDZ ELECTRA	73.9 (4.1)	73.4 (8.9)	75.1 (3.5)	66.2 (3.6)	63.3 (7.2)	70.1 (4.2)	81.6 (1.4)	80.5 (3.2)	82.9 (0.7)
GBERT	76.2 (0.9)	74.5 (3.1)	78.0 (2.0)	68.2 (4.3)	66.4 (7.4)	70.4 (1.9)	81.9 (1.0)	80.9 (2.2)	82.9 (1.2)
GELECTRA	79.9 (2.0)	78.6 (3.5)	81.4 (0.8)	78.5 (2.5)	77.6 (5.0)	**79.7 (2.5)**	83.0 (0.3)	81.3 (1.3)	84.7 (1.3)
GottBERT	79.3 (3.7)	77.5 (5.4)	81.2 (2.4)	79.8 (2.3)	80.8 (3.7)	79.1 (5.2)	**83.9 (0.3)**	82.4 (0.9)	**85.4 (0.7)**
GermanBERT	76.4 (1.2)	75.1 (4.3)	77.9 (2.9)	72.7 (3.8)	71.1 (6.9)	74.8 (3.3)	83.4 (0.3)	83.3 (0.5)	83.4 (0.5)
mBERT	62.5 (6.6)	55.9 (8.9)	71.6 (3.5)	61.4 (5.2)	56.3 (8.3)	68.5 (4.4)	79.4 (1.4)	76.9 (2.1)	82.0 (0.7)
sBERT	80.8 (1.0)	82.3 (1.6)	79.3 (1.5)	73.7 (2.0)	78.5 (2.9)	69.6 (3.8)	83.0 (0.3)	**83.8 (1.1)**	82.3 (0.7)
Bi-LSTM-CRF	78.6 (1.8)	78.5 (2.1)	78.8 (3.8)	74.8 (2.0)	76.8 (5.4)	73.2 (2.2)	79.5 (0.4)	80.9 (1.6)	78.2 (1.1)
Borchert et al.	–	–	–	–	–	–	67.7^a^	94.5^a^	52.8^a^

*Notes*: This table shows the micro-averaged scores for each model. Since nested cross-validation was performed, the mean and standard deviation are reported. The best-performing models are highlighted in bold.

a Borchert et al. evaluated their method on the entire set of manually curated text segments. In contrast, we utilized these data for nested cross-validation. As a result, the measurements cannot be directly compared and only serve to illustrate performance disparities.

For the BRONCO150 dataset, *F*_1_-scores between 46.7 and 83.2% were observed. The BioELECTRA-small, BioELECTRA-base, and mBERT models achieved the lowest performances with a gap of 36.5, 19.1, and 20.7% to the best model, respectively. All other models showed more similar performances and achieved *F*_1_-scores of 73.9–83.2%. Compared to the models Kittner et al. used, our Bi-LSTM-CRF model had a lower performance; however, the top-performing BioGottBERT model outperformed their LSTM-WE model (the authors used a bidirectional Long Short-Term Memory (LSTM) network combined with FastText word embeddings for this NER task) for all 3 entity classes: Diagnosis, Treatment, and Medication (see Supplementary Table C.1).

For the ChaDL dataset, we observed a diverse performance. The 2 BioELECTRA models performed poorly as seen on BRONCO150 dataset (61.1 and 55.3% for the small and base model, respectively). Similarly, the ClinicalBERT model, which was fine-tuned using a translated version of the ChaDL corpus (see Supplementary Material Section A.2.3 for details of the translation process), reached a low score of 44.4%. The *F*_1_-scores of the remaining models ranged between 61.4 and 80.4%, and as before, BioGottBERT scored best. The top-performing models, BioGottBERT, GottBERT, and GELECTRA, outperformed our Bi-LSTM-CRF model.

The results obtained on the GGPONC dataset are for most models in a more similar range (79.4–83.9% without BioELECTRA-base). All models except the BioELECTRA-base and mBERT outperformed the Bi-LSTM-CRF model. BioELECTRA-base achieved the lowest overall value (*F*_1_ of 65.1%); however, BioELECTRA-small achieved an *F*_1_-score of 82.3%, which came relatively close to the best value of 83.9%, achieved by GottBERT. More detailed information on the recognition of individual entities and reference metrics for all 3 datasets can be found in Supplementary Tables C.1–C.3, respectively.

Given all results, we conclude that not all transformer-based models are equally suited for biomedical and clinical applications. For the document classification tasks, we identified GBERT as the best-performing model. Our pre-trained BioGottBERT, the published GottBERT, and GELECTRA models were the best performing models for the NER tasks. In contrast to the BioGottBERT model, the newly trained BioELECTRA models proved ineffective. Except for the JSynCC dataset, the base model performed significantly worse than most other models. The small model performed well on CLEF, JSynCC, and GGPONC but was inferior for the 2 clinical datasets, BRONCO150 and ChaDL.

## DISCUSSION

Clinical notes represent a vital resource for communication between medical experts. As information hidden in clinical notes has a high potential to support medical research and clinical applications, the accurate extraction and structuring of such patient information are essential. For this purpose, novel systems are needed that are specifically designed for the clinical domain. This study addressed the applicability of publicly-available transformer-based language models for the German clinical language domain. Furthermore, we developed new biomedical models by pre-training them on a large biomedical corpus, and we systematically assessed their performances compared to 8 further GPLMs.

One contribution of this study is the development of 3 new transformer-based language models which we trained on a newly compiled corpus of biomedical text. As described in the Results section, the domain-adapted BioGottBERT achieved – in agreement with our expectations – a higher MLM accuracy than the initial GottBERT model, implying a better understanding of biomedical language. On the other hand, the pre-training of the 2 BioELECTRA models displays unexpected behavior. As described previously, the base model achieved a higher MLM accuracy than the small model. In contrast, the final RTD accuracy of the base model was much lower than the small models’, implying that the base model’s generator predicted masked tokens more accurately, complicating the discriminators’ task to differentiate original and replaced tokens. Meanwhile, the lower performance of the small models’ generator made the discriminators’ job easier.

Furthermore, we created ChaDL, a new clinical dataset for NER. We annotated 50 discharge letters with medical terms and achieved satisfactory quality according to the calculated inter-annotator agreement score. In addition, we utilized the BRONCO150, CLEF eHealth 2019, GGPONC, and JSynCC datasets. Although the nature of the datasets varies, it is helpful to use all of them in order to evaluate a broad range of biological language understanding. By using clinical and biological datasets, we followed the example of the English benchmark Biological Language Understanding Evaluation (BLUE).^50^ While the GGPONC, JSynCC, and CLEF eHealth 2019 datasets are based on clinical guidelines, fictional text, or NTPs, BRONCO150 and ChaDL are based on discharge letters and, therefore, are more important to assess the performance for clinical applications. While BRONCO150 contains more discharge letters (150 vs 50), ChaDL benefits from the integrity of the entire documents rather than single, randomly mixed sentences. Therefore, we believe that ChaDL reflects real-world clinical applications more accurately than the other datasets.

The final contribution is the systematic comparison of all mentioned models. The fine-tuning results for the 5 datasets indicated positive effects of domain adaption. BioGottBERT outperformed GottBERT on BRONCO150 and ChaDL while being only marginally inferior on the GGPONC dataset. However, the pre-training from scratch showed no positive effects for the 2 BioELECTRA models, which were strongly outperformed by all other models on the 2 clinical datasets, BRONCO150 and ChaDL. The domain-adaption’s lower environmental impact (see Supplementary Appendix B.2) provides further support for this strategy.

The overall results of this study align well with previous studies. On the one hand, it has been shown by Bressem et al.^31^ and Richter-Pechanski et al.^32^ that training from scratch led, so far, to lower performances compared to GPLMs and is, therefore, not advantageous. On the other hand, it has been shown that domain-adapted models can have improved performance compared to the initial model.^22^^,^^24^^,^^31^ For instance, Rad-BERT achieved on average a 2% higher AUC than the initial GermanBERT model on the classification of chest radiograph reports, and in the English domain using BioBERT instead of BERT on the NCBI disease dataset increased the F1 score by 1.1%.

We believe that the low performance of newly trained models is mainly due to the relatively small size of available pre-training corpora. Compared to GermanBERT, we only had about 6.7% of the data used for pre-training, and in the case of GottBERT, it was only 0.5%. Training models from scratch proved unsuccessful with such a limited amount of data. Nevertheless, we see a need to compile a larger German biomedical corpus in the near future so that the limits of German biomedical NLP models can be pushed further using domain-adaption strategies.

Aside from the encouraging results for the domain-adaption strategy, our study also confirms that GPLMs perform surprisingly well on clinical NLP tasks. In particular, GBERT achieved excellent results for the document classification tasks, while GottBERT and GELECTRA excelled for the NER tasks. Although domain-specific models will most likely outperform unspecific language models when larger corpora of biomedical texts are available, these models seem to be well suited as a first approach for conducting research when domain-specific models are unavailable. Furthermore, we found that the best transformer-based models outperformed Bi-LSTM-CRF models when applied to BRONCO150, ChaDL, and GGPONC, which demonstrates the potential of these models for the development of biomedical NLP applications.

To completely comprehend a model’s capability for clinical applications, we suggest conducting additional research to evaluate German language models on relation extraction, question answering, and named-entity normalization tasks. In this regard, it would be ideal for further gathering a diverse set of publicly available datasets for a German analog of the BLUE,^50^ allowing direct comparison of future models.

### Limitations

While conducting our work, we faced 2 main limitations: First, there is a small amount of pre-training data we acquired. Access to German clinical documents for scientists is often severely restricted if the studies are not carried out at a hospital. Similarly, biomedical data are not as abundant as in the English language. Focusing on drug leaflets, Wikipedia, and scientific abstracts, we only retrieved 0.8 GBs of textual data, which hindered the pre-training of a transformer-based model for the biomedical domains.

Second, German biomedical and clinical datasets are rare, and there is no standardized benchmark for performance assessment. As already reported in prior studies,^51^ there are large differences between English and non-English resources. While no datasets were available a couple of years ago, we now have access to 4 public datasets, BRONCO150, the CLEF eHealth dataset, GGPONC, and JSynCC. In this study, we used them alongside our dataset ChaDL for the evaluation. While all of them are suited for a performance comparison of several models, some are still subject to restrictions. JSynCC suffers from class imbalance, BRONCO150 contains some very short training samples due to the fragmentation into shuffled sentences, and ChaDL consists of relatively few clinical documents.

## CONCLUSION

In this study, we investigated the performance of both general-purpose and newly trained domain-specific transformer-based models for the German-language biomedical domain. On the one hand, our findings indicate that training new models from scratch with a small amount of biomedical data are currently ineffective and results in models that are inferior to existing models. On the other hand, we observed that previously published general-purpose models performed remarkably well on the biomedical named-entity recognition and document classification tasks. We were able to slightly enhance performances by domain-adapting an existing model, showing that the domain-adaptation strategy has potential. If larger corpora for the biomedical domain were to become accessible in the future, the boundaries of German biomedical NLP models may be pushed even further by domain adaptation.

To support future research, we have made our pre-trained BioGottBERT model available on https://huggingface.co/SCAI-BIO/bio-gottbert-base and published our code at https://github.com/SCAI-BIO/transformers-for-german-biomedical-nlp.

## FUNDING

This research was performed in the context of “KEY2AI-MED: Key Technologies for a Scalable Medical AI and Data Platform” initiative and supported by the Fraunhofer “Innopush-Program – Cross-Institute Projects” under Grant No. Anti-Corona 800081. This work has also been funded via the “COPERIMOplus” initiative and supported by the Fraunhofer “Internal Programs” under Grant No. Anti-Corona 840266.

## AUTHOR CONTRIBUTIONS

Conceptualization, supervision: SM, HF; data curation, validation: ML, VL-R; funding acquisition: MH-A, MW; methodology: ML, SM, HF; formal analysis, visualization, investigation, software: ML; resources: LK, JF, JW; project administration: SM, MJ, MH-A; writing – original draft: ML, SM; writing – review and editing: all authors.

## SUPPLEMENTARY MATERIAL

Supplementary material is available at *JAMIA Open* online.

## CONFLICT OF INTEREST STATEMENT

None declared.

## Supplementary Material

ooac087_Supplementary_Data

## Data Availability

The Charité – Universitätsmedizin Berlin provided clinical notes for the ChaDL dataset. Data access may be granted on a case-by-case basis to researchers who meet the necessary criteria for adhering to institutional data privacy policies and protocols. Please contact Joachim Weber (joachim.weber@bih-charite.de) for access. The previously published datasets BRONCO150 and GGPONC can be requested from the respective authors. JSynCC can be compiled using published code, and the CLEF eHealth dataset is freely available.
