# Supplementary material for: Critical assessment of transformer-based AI models for German clinical notes
Source: JAMIA Open. 2022 Nov 15;5(4):ooac087. doi: 10.1093/jamiaopen/ooac087 (PMC9663939; doi:10.1093/jamiaopen/ooac087)
Supplement: ooac087_Supplementary_Data [file ooac087_supplementary_data.pdf]

# A DATASETS

## A.1 Pre-Training Corpora

Supplementary Table A.1: Overview of biomedical corpora compiled for pre-training.

| | Size [MB] | Tokens | Unique Lemmata (spaCy) |
|---|---|---|---|
| AMIce drug leaflets | 86 | 7,068,760 | 75,510 |
| LIVIVO abstracts | 475 | 38,374,137 | 1,232,566 |
| Wikipedia articles (Medicine) | 248 | 20,637,029 | 1,024,539 |
| Total | 809 | 66,079,926 | 1,953,033 |

## A.2 ChaDL Dataset

The annotation process of the discharge letters was performed in two steps. First, the sections of a discharge letter were marked and classified into the following categories. Subsequently, the relevant entities were annotated within a subset of sections.

### A.2.1 Annotation of Sections

The clinical notes were divided into the following zones.

- Header and Footer
- Personal data
- Diagnoses
- Anamneses
- Medication
- Procedures and measures
- Findings
- Epicrisis

The **header** and **footer** refer to the standardized header and footer of the discharge letter. The **personal data** section follows the header and includes the patient's name, address, admission date, and discharge date. We annotated none of these three sections any further.

The most important sections for the annotation process were the Diagnoses, Anamneses, Medication, and Epicrisis sections. The **diagnosis section** usually consists of a list containing diagnoses and performed procedures. The **anamnesis section** is typically free text and contains detailed descriptions of the patient's condition. The **medication section** is usually in list form. It includes the medications, dosage, and intake order (usually according to the morning-noon-evening-night schedule). Depending on the

report, there may be a section for medication on admission and discharge. In addition to the anamnesis section, the **epicrisis** is essential. This is free text, usually describing the entire treatment with key outcomes.

Besides the already mentioned sections, we annotated the procedures, measures, and findings sections. **procedures and measures** does not necessarily occur in every discharge letter. This section mainly includes a listing of caretakers' actions (e.g., change of bandages). The **findings section** primarily includes findings (e.g., from laboratory tests or computed tomography) in a list table or list form. Since both sections were often poorly formatted and did not contain much text, we did not annotate them further.

### A.2.2 Annotation of Entities

After the annotation of sections, the relevant entities were annotated in the sections medication, diagnoses, anamneses, and epicrisis. We restricted ourselves to these sections since their annotation was easiest due to simpler structuring, and they contain the greatest amount of information.

Marked entities included medications (**MEDICATION**), dose information (**DOSAGE**), and medication orders (**INTAKE**). Under the term medication, everything that was administered to the patient to improve the symptoms was recorded. For this purpose, dose information such as mg, IU, or $\mu l$ was marked. Medication orders were mostly documented according to the "morning-noon-evening-night" (e.g., 1-0-0-0) scheme, but free text such as "over 12 months" was also marked in addition to this information.

Furthermore, we marked disorders (**DISORDER**). That includes anything that contributes to disease, meaning that, e.g., symptoms like fever or cough and the diseases such as influenza or diabetes mellitus type 2 are covered by this term.

Finally, therapeutic (**PROCEDURES**) and diagnostic measures (**DIAGNOSTICS**) were annotated. The former includes everything that, for instance, caretakers did to improve the patient's condition. Examples are diuretic therapy and anti-aggregation therapy. The latter include those undertaken to establish a diagnosis or record the current status. Examples are MRI or continuous ECG.

The following bullet points describe occurring scenarios and our annotation guideline in more detail:

- In all cases, we annotated the largest possible text span that describes a disease or drug in the best way. For instance, we did not only annotate **infarct** but rather **cerebral infarct** or **diabetes mellitus type 2** instead of **diabetes mellitus**.
- Successive terms which depend on each other should be annotated together. For instance: **DM (diabetes mellitus)** and **Zocor (Simvastatin)**
- Enumerations were annotated separately. In the example *[...] headache, fever, and vomiting [...]* each term was annotated individually.
- In some cases, dosage information is unclear (e.g., for infusions), so we did not annotate it.

One example is shown in the following figure:

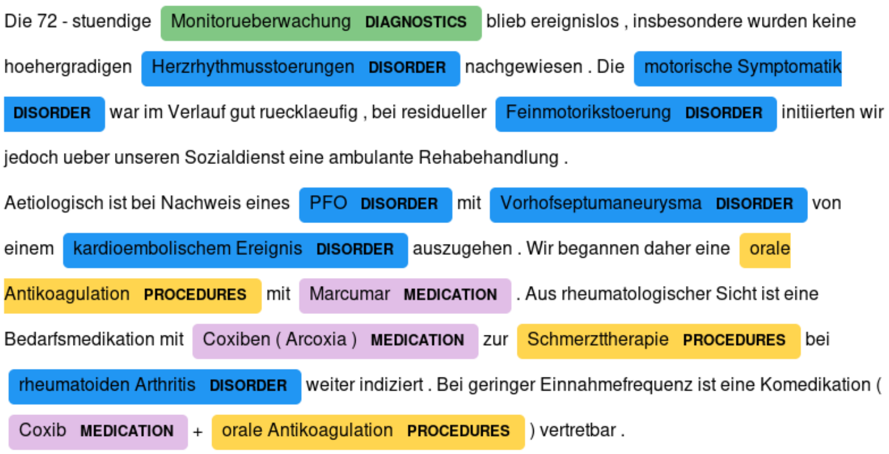


Supplementary Figure A.1: **Exemplary excerpt from the ChaDL corpus.**

### A.2.3 Translation of ChaDL

As part of our experiments with ChaDL, we assessed how a model trained on English clinical data, ClinicalBERT, performs on a translated version of our ChaDL corpus. We used the Marian framework (version 1.10.0, [1]) for neural machine translation and the German-to-English model from the Tatoeba challenge, which was trained on the OPUS corpus (`https://github.com/Helsinki-NLP/Tatoeba-Challenge/tree/master/models/deu-eng`, accessed on 23 December 2021, released on 30 April 2021). The model achieved a BLEU score of 40.3 and 53.1 on the *newstest2019* and *Tatoeba* datasets (further metrics can be found at the above-mentioned Github repository). The original annotations were mapped to the translated text based on sentence alignments. These alignments were created using the *SentenceAligner* from the *simalign* python package and the mBERT model.[2] The following shows one example of a German clinical note and its English translation.

| | |
|---|---|
| Epikrise und Beurteilung : Die Übernahme der kardiopulmonal stabilen Patientin erfolgte nach STEMI aus unserer kardiologischen Intensivstation zur weiteren Überwachung und Therapie . Die CK stieg maximal auf 1733 / 205 U / l und war im Verlauf regredient . Zuletzt sahen wir die CK im Normbereich bei asymptomatischer Patientin . In der telemetrischen Überwachung konnten keine höhergradihen Herzrhythmusstörungen detektiert werden . | Epicrisis and assessment : The takeover of the cardiopulmonary stable patient took place according to STEMI from our cardiological intensive care unit for further monitoring and therapy. The CK rose to a maximum of 1733 / 205 U / l and was regredient during the course. Most recently we saw the CK in the normal range in asymptomatic patient. In telemetric monitoring no higher degrees of cardiac arrhythmia could be detected. |

## A.3 Public Datasets

The following figures show excerpts of the freely available datasets BRONCO150, GGPONC, and JSyncc. The former two can be requested from the authors, and the code for the generation of the latter is available via GitHub.

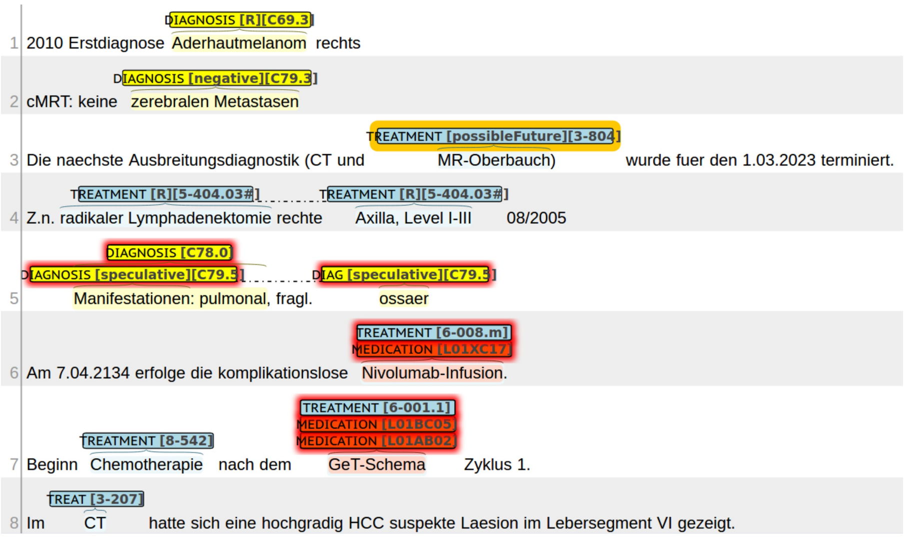


Supplementary Figure A.2: **Exemplary excerpt from the BRONCO corpus.** [3]

```
<document>
 <section>
  <name>Risikofaktoren</name>
  <section>
   <name>Helicobacter pylori</name>
   <recommendation>
    <recommendation_creation_date
       value="2019-01-01"/>
    <recommendation_grade value="B"/>
    <!-- more metadata -->
    <text>Die H. pylori-Eradikation
       mit dem Ziel der Magenkarzinom
       -prävention sollte bei den
       folgenden Risikopersonen
       durchgeführt werden (siehe
       Tabelle unten).</text>
   </recommendation>
   <text>Das Magenkarzinom ist eine
      multifaktorielle Erkrankung,
      bei der die Infektion mit H.
      pylori den wichtigsten
      Risikofaktor darstellt. Seit
      1994 ist H. pylori durch die
      Weltgesund-heitsorganisation
      als Klasse I Karzinogen
      anerkannt und wurde 2009 als
      solches bestätigt <litref id="
      65327"/>.</text>
  </section> <!-- more sections -->
 </section>
</document> <!-- more documents -->
```

Supplementary Figure A.3: **Exemplary excerpt from the GGPONC corpus.** [4]

```xml
<?xml version="1.0" encoding="UTF-8" standalone="yes"?>
<corpus>
  <document>
    <id>1</id>
    <text>Vorgeschichte/Indikation: Sturz auf den
      Schädel unter Alkoholeinfluss. Anschl.
      HWS-Schmerzen. Konventionell radiologisch sowie
      im CT Nachweis der u.g. Fraktur. (...)</text>
    <type>operation report</type>
    <heading>Densfraktur - Verschraubung</heading>
    <topic>Orthopädie</topic>
    <topic>Unfallchirurgie</topic>
    <source>Siekmann, H., Irlenbusch, L., and Klima, S.
      (2016). Operationsberichte Orthopädie und
      Unfallchirurgie. Springer-Verlag.</source>
  </document>  (...)
</corpus>
```

Supplementary Figure A.4: **Exemplary excerpt from the JSynCC corpus.** [5]

## A.4 Class Frequency of JSynCC Corpus

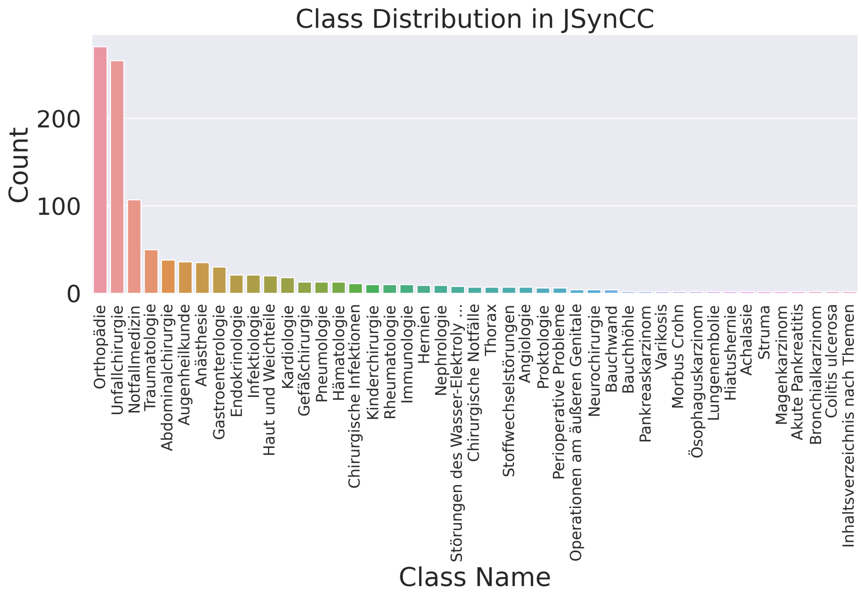


Supplementary Figure A.5: **Class Distribution in JSynCC.** Shown is the count for most classes in the JSynCC corpus. Unique classes were excluded for visualization purposes.

# B TRAINING

## B.1 Pre-Training Hyperparameters

Supplementary Table B.1: Pre-Training Parameters

| Name | BioELECTRA-small | BioELECTRA-base | BioGottBERT |
|---|---|---|---|
| Batch size | 128 | 336 | 2016 |
| Hidden size | 256 | 768 | 768 |
| FNN inner hidden size | 1024 | 3072 | 3072 |
| Attention heads | 4 | 12 | 12 |
| Attention head size | 64 | 64 | 64 |
| Embedding size | 128 | 768 | 768 |
| Generator size | 1/4 | 1/3 | – |
| Mask percent | 0.15 | 0.15 | 0.15 |
| Warmup steps | 10K | 10K | 2K |
| Learning rate | 5e-4 | 2e-4 | 4e-4 |
| Adam $\epsilon$ | 1e-6 | 1e-6 | 1e-6 |
| Adam $\beta_1$ | 0.9 | 0.9 | 0.9 |
| Adam $\beta_2$ | 0.999 | 0.999 | 0.999 |
| Dropout | 0.1 | 0.1 | 0.1 |
| Weight decay | 0.01 | 0.01 | 0.01 |
| Train steps | 1M | 580K | 50K |

## B.2 Hyperparameter Optimization for Fine-Tuning

This section lists the hyperparameters that were optimized in our experiments. We performed Bayesian hyperparameter optimization to tune the parameters for the Transformer-based models. We used a Tree-structured Parzen Estimator (TPE)[6]; the parameter ranges are listed in the following table.

Supplementary Table B.2: Optimized Hyperparameters for Transformer-based Models.

| Name | Values/Range |
|---|---|
| Batch size | 8, 16, 32 |
| Learning rate | $[1 \cdot 10^{-5}, 5 \cdot 10^{-4}]$ |
| Weight decay | $[1 \cdot 10^{-10}, 1 \cdot 10^{-1}]$ |

Since the experiments for the Bi-LSTM+CRF models are more time-consuming than the ones with the Transformer-based models, we performed a more straightforward grid search with the following parameters.

Supplementary Table B.3: Hyperparameters for Bi-LSTM+CRF Model. The parameters which were changed during the grid search are written in square brackets.

| Name | Values/Range |
|---|---|
| Anneal Factor | 0.5 |
| Batch size | [8, 16, 32] |
| Hidden size | 256 |
| Learning rate | 0.1 |
| Patience | 3 |
| RNN Layers | [1, 2, 3] |

## Environmental Impact of Model Pre-training

We used the Green Algorithms [7] tool to estimate the environmental impact of our pre-trained models. Therefore we provided the resources and time required to train our models.

Supplementary Table B.4: **Estimated CO2 emissions for model pre-training.** We estimated the CO2 emissions with Green Algorithms (`http://www.green-algorithms.org/`, accessed on 08.07.2022) based on the required resources and the duration of training.

| Model | Hours trained | GPUs | CPUs | kWh | kg CO2e |
|---|---|---|---|---|---|
| BioELECTRA-small | 193.0 | 2 P100 | 8 | 199.78 | 67.66 |
| BioELECTRA-base | 384.5 | 4 P100 | 8 | 719.07 | 243.52 |
| BioGottBERT | 94.0 | 4 A100 | 8 | 175.79 | 59.53 |

# C FINE-TUNING PERFORMANCE

## C.1 Entity-wise Results

Supplementary Table C.1: **Entity-wise F1 Scores for the BRONCO150 dataset.** Entity-wise F1 scores for each model. In addition to the evaluated Transformer-based models, the reference scores from Kittner et al. are reported. The best-performing models are highlighted in bold.

| *Model* | Diagnosis | Medication | Treatment | Micro F1 |
|---|---|---|---|---|
| BioELECTRA-base | 69.9 (2.6) | 45.7 (13.9) | 77.8 (3.4) | 64.1 (6.0) |
| BioELECTRA-small | 54.8 (14.8) | 31.0 (2.8) | 52.9 (14.4) | 46.7 (10.3) |
| BioGottBERT | **78.1 (2.3)** | **94.0 (1.4)** | **84.9 (1.7)** | **83.2 (1.6)** |
| DBMDZ ELECTRA | 68.5 (4.9) | 84.7 (6.8) | 76.2 (2.9) | 73.9 (4.1) |
| GBERT | 71.0 (1.2) | 88.3 (2.4) | 77.7 (2.5) | 76.2 (0.9) |
| GELECTRA | 76.3 (1.8) | 90.5 (2.6) | 79.8 (2.4) | 79.9 (2.0) |
| GottBERT | 75.3 (2.3) | 92.3 (0.9) | 78.8 (8.9) | 79.3 (3.7) |
| germanBERT | 70.8 (1.2) | 90.0 (1.1) | 77.6 (2.0) | 76.4 (1.2) |
| mBERT | 62.8 (3.4) | 70.0 (11.3) | 59.2 (10.4) | 62.5 (6.6) |
| sBERT | 75.6 (0.8) | 92.6 (1.7) | 82.3 (2.2) | 80.8 (1.0) |
| Bi-LSTM-CRF | 73.6 (1.9) | 90.4 (0.9) | 79.8 (3.1) | 78.6 (1.8) |
| CRF | 75 (2) | 90 (1) | 82 (1) | – |
| CRF + WE | 74 (1) | 90 (1) | 81 (1) | – |
| LSTM | 72 (1) | 88 (2) | 81 (2) | – |
| LSTM + WE | 77 (8) | 91 (4) | 84 (6) | – |

Supplementary Table C.2: **Entity-wise F1 Scores for ChaDL.** Entity-wise F1 scores and the standard deviation of the results obtained by the nested cross-validation. The best results are highlighted in bold.

| *Model* | Diagnostics | Disorder | Dosage | Intake | Medication | Procedures | Micro F1 |
|---|---|---|---|---|---|---|---|
| BioELECTRA-base | 56.1 (10.6) | 60.5 (14.2) | 66.8 (27.2) | 65.2 (20.3) | 46.0 (13.4) | 59.3 (6.8) | 55.3 (10.5) |
| BioELECTRA-small | 62.0 (3.2) | 69.9 (4.2) | 80.6 (11.4) | 51.0 (9.6) | 50.5 (18.2) | 64.7 (5.2) | 61.1 (6.5) |
| BioGottBERT | **73.2 (4.1)** | **77.8 (2.2)** | 90.7 (5.8) | 84.5 (5.4) | 88.3 (4.1) | **75.3 (7.3)** | **80.4 (1.1)** |
| ClinicalBERT | 35.5 (8.4) | 40.6 (4.2) | 72.4 (14.8) | 65.8 (8.6) | 47.4 (17.6) | 34.6 (8.6) | 44.4 (6.0) |
| DBMDZ ELECTRA | 64.8 (2.8) | 68.1 (4.3) | 51.8 (19.3) | 71.7 (8.4) | 81.6 (4.9) | 61.5 (7.6) | 66.2 (3.6) |
| GBERT | 67.1 (5.5) | 69.1 (2.3) | 72.7 (21.6) | 67.4 (8.6) | 70.5 (8.1) | 63.3 (4.3) | 68.2 (4.3) |
| GELECTRA | 71.0 (6.3) | 76.0 (3.4) | 90.2 (5.7) | 82.0 (5.0) | 87.5 (3.3) | 71.0 (3.9) | 78.5 (2.5) |
| GottBERT | 72.9 (2.3) | 76.2 (3.7) | **91.4 (6.2)** | **86.3 (5.0)** | **88.8 (5.1)** | 74.2 (5.7) | 79.8 (2.3) |
| germanBERT | 65.2 (4.3) | 70.8 (3.1) | 79.3 (14.5) | 73.6 (7.8) | 83.2 (6.3) | 66.5 (2.2) | 72.7 (3.8) |
| mBERT | 62.7 (7.1) | 63.2 (4.5) | 60.2 (16.7) | 69.9 (15.3) | 59.4 (8.5) | 57.1 (9.9) | 61.4 (5.2) |
| sBERT | 68.9 (5.4) | 68.1 (2.6) | 89.9 (6.9) | 84.1 (6.4) | 84.7 (6.6) | 64.7 (6.3) | 73.7 (2.0) |
| Bi-LSTM-CRF | 65.8 (5.1) | 70.6 (0.9) | 86.8 (7.5) | 84.7 (4.6) | 86.9 (5.9) | 65.3 (5.8) | 74.8 (2.0) |

Supplementary Table C.3: **Entity-wise F1 Scores for GGPONC.** Entity-wise F1 scores for each entity. The best results are highlighted in bold.

| *Model* | Anatomical Structure | Chemicals Drugs | Devices | Disorders | Living Beings | Physiology | Procedures | TNM | Micro F1 |
|---|---|---|---|---|---|---|---|---|---|
| BioELECTRA-base | 70.6 (6.6) | 62.0 (10.0) | 51.0 (7.8) | 56.8 (15.4) | 90.6 (3.7) | 51.6 (7.1) | 70.8 (7.0) | 77.1 (7.0) | 65.1 (7.3) |
| BioELECTRA-small | 79.8 (1.7) | 88.6 (1.3) | 56.9 (6.6) | 83.9 (0.5) | 93.9 (0.4) | 62.5 (2.2) | 78.7 (0.7) | 87.0 (3.1) | 82.3 (0.4) |
| BioGottBERT | **81.9 (1.2)** | 89.6 (0.6) | **62.6 (3.6)** | 85.0 (1.4) | 94.2 (0.4) | **64.6 (1.5)** | **80.8 (0.3)** | 89.7 (2.0) | 83.8 (0.4) |
| DBMDZ ELECTRA | 79.5 (1.3) | 88.4 (1.6) | 59.7 (5.0) | 82.8 (2.3) | 93.8 (0.3) | 60.2 (1.9) | 78.0 (1.6) | 89.3 (1.8) | 81.6 (1.4) |
| GBERT | 77.4 (1.5) | 87.4 (2.4) | 59.4 (7.0) | 83.8 (0.7) | 93.3 (0.8) | 61.8 (1.6) | 78.6 (1.7) | 87.6 (2.6) | 81.9 (1.0) |
| GELECTRA | 81.4 (1.7) | 89.1 (0.4) | 60.5 (1.6) | 85.3 (0.8) | 94.0 (0.5) | 62.8 (1.1) | 79.3 (1.0) | 89.4 (4.4) | 83.0 (0.3) |
| GottBERT | **81.9 (1.4)** | **89.7 (0.5)** | 60.6 (7.8) | **86.0 (0.3)** | **94.5 (0.3)** | 63.2 (2.1) | 80.6 (0.7) | 90.3 (1.1) | **83.9 (0.3)** |
| germanBERT | 81.0 (2.0) | 89.3 (0.6) | 61.3 (4.6) | 84.9 (1.0) | 94.1 (0.4) | 63.5 (1.4) | 80.0 (0.6) | **90.7 (3.0)** | 83.4 (0.3) |
| mBERT | 75.9 (2.6) | 86.2 (1.4) | 57.7 (11.0) | 81.1 (2.0) | 91.8 (3.4) | 59.9 (3.6) | 75.3 (1.7) | 85.8 (4.7) | 79.4 (1.4) |
| sBERT | 79.3 (1.4) | 88.8 (0.3) | 60.8 (2.8) | 84.9 (0.7) | 94.1 (0.4) | 62.5 (3.4) | 79.6 (0.5) | 88.7 (2.0) | 83.0 (0.3) |
| Bi-LSTM-CRF | 77.5 (1.2) | 83.9 (0.6) | 52.0 (6.6) | 81.8 (0.7) | 93.1 (0.4) | 56.4 (3.0) | 75.7 (0.8) | 85.5 (4.6) | 79.5 (0.4) |

Supplementary Table C.4: **Class-wise F1 Scores for JSynCC (version B).** Average F1 scores for each class. The best results are highlighted in bold.

| *Model* | Orthopedics | Accident surgery | Emergency medicine | Traumatology | Micro F1 |
|---|---|---|---|---|---|
| BioELECTRA-base | 90.6 (2.2) | 87.6 (2.0) | 99.6 (1.0) | 96.0 (4.6) | 91.2 (1.6) |
| BioELECTRA-small | 90.5 (2.4) | 87.4 (2.0) | 96.4 (8.1) | 90.3 (13.8) | 90.3 (1.7) |
| BioGottBERT | 88.5 (3.3) | 84.9 (2.2) | 99.3 (1.7) | 92.6 (8.2) | 89.0 (1.9) |
| DBMDZ ELECTRA | 90.3 (3.4) | 88.0 (2.8) | **100.0 (0.0)** | 98.8 (2.6) | 91.4 (2.0) |
| GBERT | **92.6 (4.5)** | **89.0 (2.9)** | 99.6 (1.0) | 97.8 (5.0) | **92.7 (2.3)** |
| GELECTRA | 90.4 (3.8) | 87.6 (2.8) | **100.0 (0.0)** | **100.0 (0.0)** | 91.4 (1.3) |
| GottBERT | 88.2 (4.2) | 85.1 (3.6) | **100.0 (0.0)** | 97.0 (4.4) | 89.5 (2.6) |
| germanBERT | 91.7 (3.4) | 87.3 (3.1) | 99.6 (1.0) | **100.0 (0.0)** | 91.8 (2.0) |
| mBERT | 90.8 (2.7) | **89.0 (3.0)** | **100.0 (0.0)** | 96.8 (7.1) | 91.9 (1.7) |
| sBERT | 88.8 (3.7) | 86.7 (3.2) | **100.0 (0.0)** | 98.0 (2.8) | 90.3 (2.1) |
| Bi-LSTM-CRF | 90.4 (2.4) | 86.7 (11.7) | **100.0 (0.0)** | **100.0 (0.0)** | 91.3 (3.6) |

## C.2 Additional Experiments With the JSynCC Dataset

In addition to the experiments described in the main article, we performed experiments with the nearly complete JSynCC dataset. We only kept class labels which occurred at least five times, which reduced the number of documents from 867 to 849. Supplementary Table C.6 shows a detailed comparison of the two JSynCC subsets used in this study.

These fine-tuning experiments were performed as described in the methods section. The results are depicted in Supplementary Table C.5. GBERT, BioGottBERT, and GermanBERT obtained the highest micro-averaged F1 scores. Compared to the performance of the Bi-LSTM-CRF model, all transformer-based models performed better. However, the scores were significantly lower than in our experiments with JSynCC version B, for which we kept only the four classes with more than 50 training samples (81.1 % vs. 92.7 %). In our experiments with version A, we obtained F1 scores of 0 % for many classes, which is not surprising given the low number of samples available for training and evaluation. Therefore, we conclude that transformer-based models are well suited for biomedical document classification tasks, but only if an adequate amount of data for the respective classes are available.

Supplementary Table C.5: **Overview of the JSynCC dataset**. This table provides detailed information about the number of documents, sentences, tokens, and documents per class for both versions of the JSynCC dataset that we used in this study.

| | JSynCC | |
|---|---|---|
| | version A | version B |
| *Textual elements* | | |
| Documents/Segments | 849 | 494 |
| Sentences | 24,985 | 20,971 |
| Tokens | 337,925 | 275,700 |
| *Document classes* | | |
| Accident surgery | | 266 |
| Emergency medicine | | 107 |
| Orthopedics | | 282 |
| Traumatology | | 50 |
| Abdominal Surgery | 38 | – |
| Ophthalmology | 36 | – |
| Anesthesia | 35 | – |
| Gastroenterology | 30 | – |
| Infectiology | 21 | – |
| Endocrinology | 21 | – |
| Skin and soft tissues | 20 | – |
| Cardiology | 18 | – |
| Pneumology | 13 | – |
| Hematology | 13 | – |
| Vascular surgery | 13 | – |
| Surgical infections | 11 | – |
| Pediatric surgery | 10 | – |
| Rheumatology | 10 | – |
| Immunology | 10 | – |
| Nephrology | 9 | – |
| Hernias | 9 | – |
| Disturbances (water-electrolyte/acid-base balance) | 8 | – |
| Surgical emergencies | 7 | – |
| Thorax | 7 | – |
| Metabolic disorders | 7 | – |
| Angiology | 7 | – |
| Proctology | 6 | – |
| Perioperative problems | 6 | – |

Supplementary Table C.6: **Class-wise F1 Scores for JSynCC (version A).** Average F1 scores for each class. The best results are highlighted in bold.

| | Bi-LSTM-CRF | BioELECTRA-base | BioELECTRA-small | BioGottBERT | DBMDZ ELECTRA | GBERT | GELECTRA | GottBERT | GermanBERT | mBERT | sBERT |
|---|---|---|---|---|---|---|---|---|---|---|---|
| Orthopedics | 91.0 (4.4) | **93.1 (5.6)** | 89.8 (3.3) | 90.5 (4.0) | 88.5 (3.8) | 92.2 (4.3) | 90.4 (4.5) | 89.9 (5.1) | 91.4 (4.8) | 90.6 (4.0) | 89.1 (3.2) |
| Accident surgery | **88.3 (3.0)** | 87.4 (2.4) | 83.9 (4.6) | 86.4 (3.4) | 84.6 (4.6) | 83.7 (2.1) | 86.0 (3.3) | 80.6 (2.4) | 87.8 (1.7) | 85.2 (4.8) | 82.4 (3.7) |
| Emergency medicine | 86.0 (7.1) | 98.9 (1.5) | 98.6 (1.3) | 99.4 (1.3) | 98.6 (1.3) | **100.0 (0.0)** | 99.5 (1.2) | 99.3 (1.6) | 99.5 (1.2) | 98.9 (2.4) | 98.4 (3.7) |
| Traumatology | 0.0 (0.0) | 77.7 (8.0) | 56.5 (34.6) | 78.4 (10.8) | 80.9 (7.6) | **83.7 (6.6)** | 43.1 (41.0) | 62.2 (35.7) | 79.4 (10.1) | 63.2 (22.4) | 81.7 (8.8) |
| Abdominal Surgery | 0.0 (0.0) | 43.4 (28.8) | 10.7 (23.9) | 77.6 (10.6) | 8.6 (19.2) | 76.7 (15.4) | 3.6 (8.1) | 45.2 (42.1) | **79.9 (15.8)** | 17.1 (23.5) | 2.4 (5.3) |
| Ophthalmology | 73.7 (42.4) | 98.8 (2.6) | **100.0 (0.0)** | **100.0 (0.0)** | 98.9 (2.4) | **100.0 (0.0)** | 96.0 (8.9) | 76.7 (43.1) | **100.0 (0.0)** | **100.0 (0.0)** | 97.8 (5.0) |
| Anesthesia | 6.7 (14.9) | 96.7 (4.9) | 96.8 (4.4) | 96.0 (5.6) | 98.7 (3.0) | **100.0 (0.0)** | **100.0 (0.0)** | 78.9 (44.2) | **100.0 (0.0)** | **100.0 (0.0)** | 77.8 (43.7) |
| Gastroenterology | 0.0 (0.0) | 14.2 (23.7) | 10.0 (22.4) | 66.5 (17.0) | 0.0 (0.0) | **67.3 (14.4)** | 0.0 (0.0) | 0.0 (0.0) | 46.1 (42.8) | 14.5 (32.5) | 0.0 (0.0) |
| Infectiology | 0.0 (0.0) | 6.7 (14.9) | 0.0 (0.0) | **37.6 (41.9)** | 0.0 (0.0) | 23.4 (32.1) | 0.0 (0.0) | 0.0 (0.0) | 16.9 (23.2) | 0.0 (0.0) | 0.0 (0.0) |
| Endocrinology | 0.0 (0.0) | 0.0 (0.0) | 0.0 (0.0) | **30.0 (29.8)** | 0.0 (0.0) | 8.1 (11.2) | 0.0 (0.0) | 0.0 (0.0) | 16.0 (21.9) | 0.0 (0.0) | 0.0 (0.0) |
| Skin and soft tissues | 0.0 (0.0) | 55.8 (32.4) | 5.7 (12.8) | 54.8 (32.0) | 8.0 (17.9) | **60.5 (38.3)** | 0.0 (0.0) | 7.3 (16.3) | 29.4 (32.7) | 16.0 (35.8) | 0.0 (0.0) |
| Cardiology | 0.0 (0.0) | 0.0 (0.0) | 0.0 (0.0) | **29.8 (27.4)** | 0.0 (0.0) | 28.9 (29.0) | 0.0 (0.0) | 0.0 (0.0) | 10.0 (22.4) | 0.0 (0.0) | 0.0 (0.0) |
| Pneumology | 0.0 (0.0) | 0.0 (0.0) | 0.0 (0.0) | 16.0 (35.8) | 0.0 (0.0) | **48.4 (33.2)** | 0.0 (0.0) | 0.0 (0.0) | 16.0 (35.8) | 0.0 (0.0) | 0.0 (0.0) |
| Hematology | 0.0 (0.0) | 16.0 (35.8) | 0.0 (0.0) | 13.3 (29.8) | 0.0 (0.0) | **36.7 (34.2)** | 0.0 (0.0) | 0.0 (0.0) | 13.3 (29.8) | 0.0 (0.0) | 0.0 (0.0) |
| Vascular surgery | 0.0 (0.0) | 0.0 (0.0) | 0.0 (0.0) | **33.3 (31.2)** | 0.0 (0.0) | 31.4 (28.8) | 0.0 (0.0) | 0.0 (0.0) | 8.0 (17.9) | 0.0 (0.0) | 0.0 (0.0) |
| Surgical infections | 0.0 (0.0) | 0.0 (0.0) | 0.0 (0.0) | 0.0 (0.0) | 0.0 (0.0) | 0.0 (0.0) | 0.0 (0.0) | 0.0 (0.0) | 0.0 (0.0) | 0.0 (0.0) | 0.0 (0.0) |
| Pediatric surgery | 0.0 (0.0) | 0.0 (0.0) | 0.0 (0.0) | 0.0 (0.0) | 0.0 (0.0) | 23.3 (32.5) | 0.0 (0.0) | 0.0 (0.0) | **53.3 (50.6)** | 0.0 (0.0) | 0.0 (0.0) |
| Rheumatology | 0.0 (0.0) | 8.0 (17.9) | 0.0 (0.0) | 8.0 (17.9) | 0.0 (0.0) | **39.4 (42.1)** | 0.0 (0.0) | 0.0 (0.0) | 28.0 (43.8) | 0.0 (0.0) | 0.0 (0.0) |
| Immunology | 0.0 (0.0) | 8.0 (17.9) | 0.0 (0.0) | 8.0 (17.9) | 0.0 (0.0) | **39.4 (42.1)** | 0.0 (0.0) | 0.0 (0.0) | 28.0 (43.8) | 0.0 (0.0) | 0.0 (0.0) |
| Nephrology | 0.0 (0.0) | 0.0 (0.0) | 0.0 (0.0) | **20.0 (44.7)** | 0.0 (0.0) | **20.0 (44.7)** | 0.0 (0.0) | 0.0 (0.0) | 0.0 (0.0) | 0.0 (0.0) | 0.0 (0.0) |
| Hernias | 0.0 (0.0) | 33.3 (47.1) | 0.0 (0.0) | 40.0 (54.8) | 0.0 (0.0) | **76.0 (14.6)** | 0.0 (0.0) | 20.0 (44.7) | 46.7 (44.7) | 13.3 (29.8) | 0.0 (0.0) |
| Disturbances (water-electrolyte & acid-base balance) | 0.0 (0.0) | 0.0 (0.0) | 0.0 (0.0) | 0.0 (0.0) | 0.0 (0.0) | 0.0 (0.0) | 0.0 (0.0) | 0.0 (0.0) | 0.0 (0.0) | 0.0 (0.0) | 0.0 (0.0) |
| Surgical emergencies | 0.0 (0.0) | 0.0 (0.0) | 0.0 (0.0) | 0.0 (0.0) | 0.0 (0.0) | 0.0 (0.0) | 0.0 (0.0) | 0.0 (0.0) | 0.0 (0.0) | 0.0 (0.0) | 0.0 (0.0) |
| Thorax | 0.0 (0.0) | 0.0 (0.0) | 0.0 (0.0) | 0.0 (0.0) | 0.0 (0.0) | **20.0 (44.7)** | 0.0 (0.0) | 0.0 (0.0) | 0.0 (0.0) | 0.0 (0.0) | 0.0 (0.0) |
| Metabolic disorders | 0.0 (0.0) | 0.0 (0.0) | 0.0 (0.0) | 0.0 (0.0) | 0.0 (0.0) | 0.0 (0.0) | 0.0 (0.0) | 0.0 (0.0) | 0.0 (0.0) | 0.0 (0.0) | 0.0 (0.0) |
| Angiology | 0.0 (0.0) | 0.0 (0.0) | 0.0 (0.0) | 0.0 (0.0) | 0.0 (0.0) | 0.0 (0.0) | 0.0 (0.0) | 0.0 (0.0) | 0.0 (0.0) | 0.0 (0.0) | 0.0 (0.0) |
| Proctology | 0.0 (0.0) | 0.0 (0.0) | 0.0 (0.0) | 0.0 (0.0) | 0.0 (0.0) | 0.0 (0.0) | 0.0 (0.0) | 0.0 (0.0) | 0.0 (0.0) | 0.0 (0.0) | 0.0 (0.0) |
| Perioperative problems | 0.0 (0.0) | 0.0 (0.0) | 0.0 (0.0) | 0.0 (0.0) | 0.0 (0.0) | 0.0 (0.0) | 0.0 (0.0) | 0.0 (0.0) | 0.0 (0.0) | 0.0 (0.0) | 0.0 (0.0) |
| Micro F1 | 70.9 (2.5) | 77.9 (2.2) | 74.7 (1.8) | 80.5 (1.3) | 75.0 (3.1) | **81.1 (1.6)** | 74.8 (4.0) | 73.6 (1.4) | 80.4 (3.3) | 75.5 (3.0) | 74.0 (2.0) |

## C.3 Relationship Between Entity Frequency and Performance

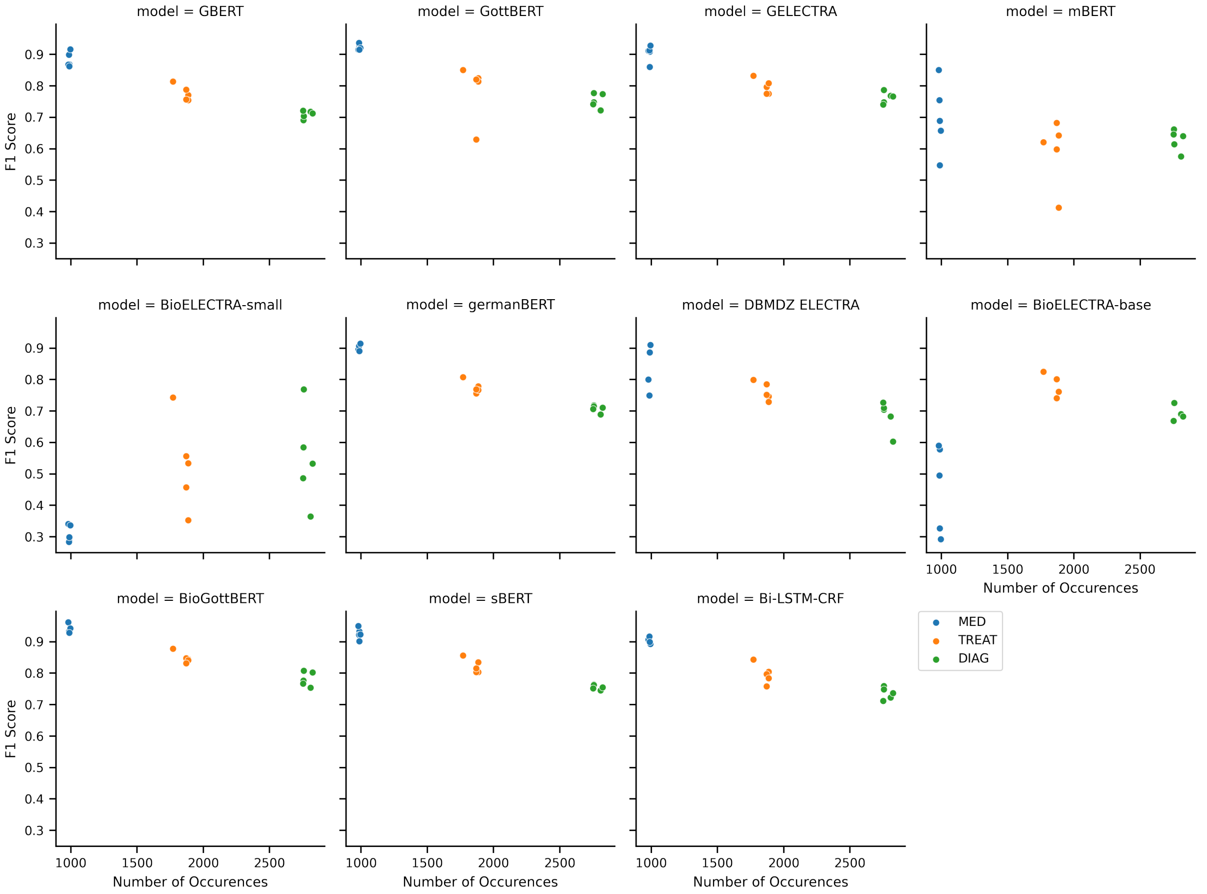


Supplementary Figure C.1: **Relationship between entity frequency and NER performance for BRONCO150.** Shown are the F1-scores for each entity with respect to the number of entities. Since nested cross-validation, we depict the results for each fold individually.

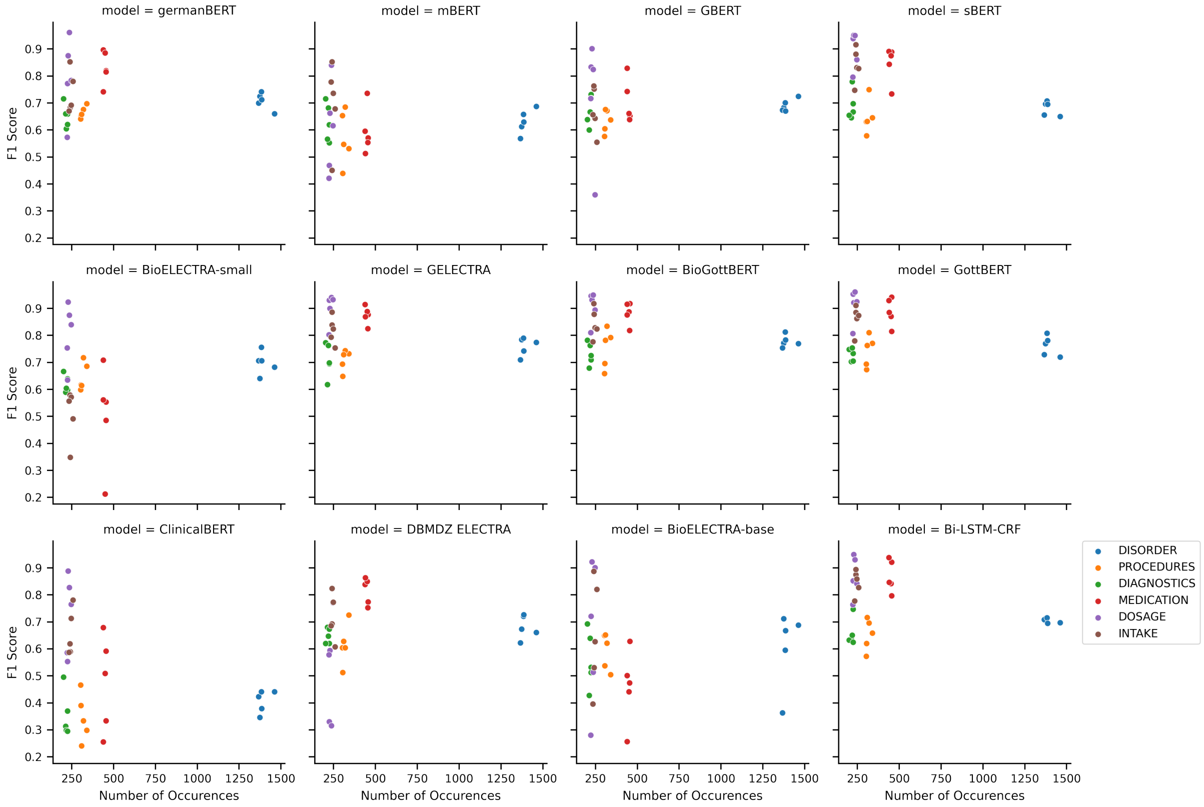


Supplementary Figure C.2: **Relationship between entity frequency and NER performance for ChaDL.** Shown are the F1-scores for each entity with respect to the number of entities. Since nested cross-validation, we depict the results for each fold individually.

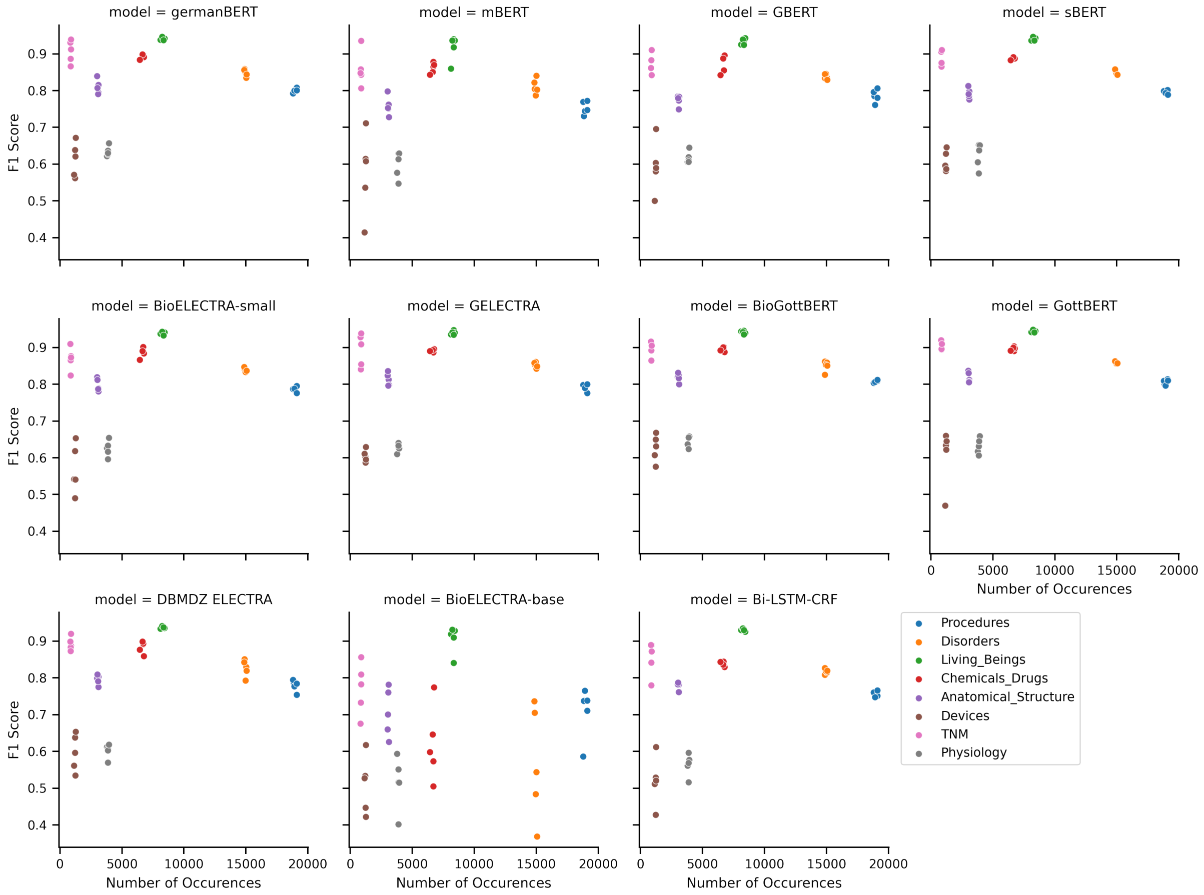


Supplementary Figure C.3: **Relationship between entity frequency and NER performance for GGPONC.** Shown are the F1-scores for each entity with respect to the number of entities. Since nested cross-validation, we depict the results for each fold individually.

## C.4 Relationship Between Pre-training Corpus Size and Performance

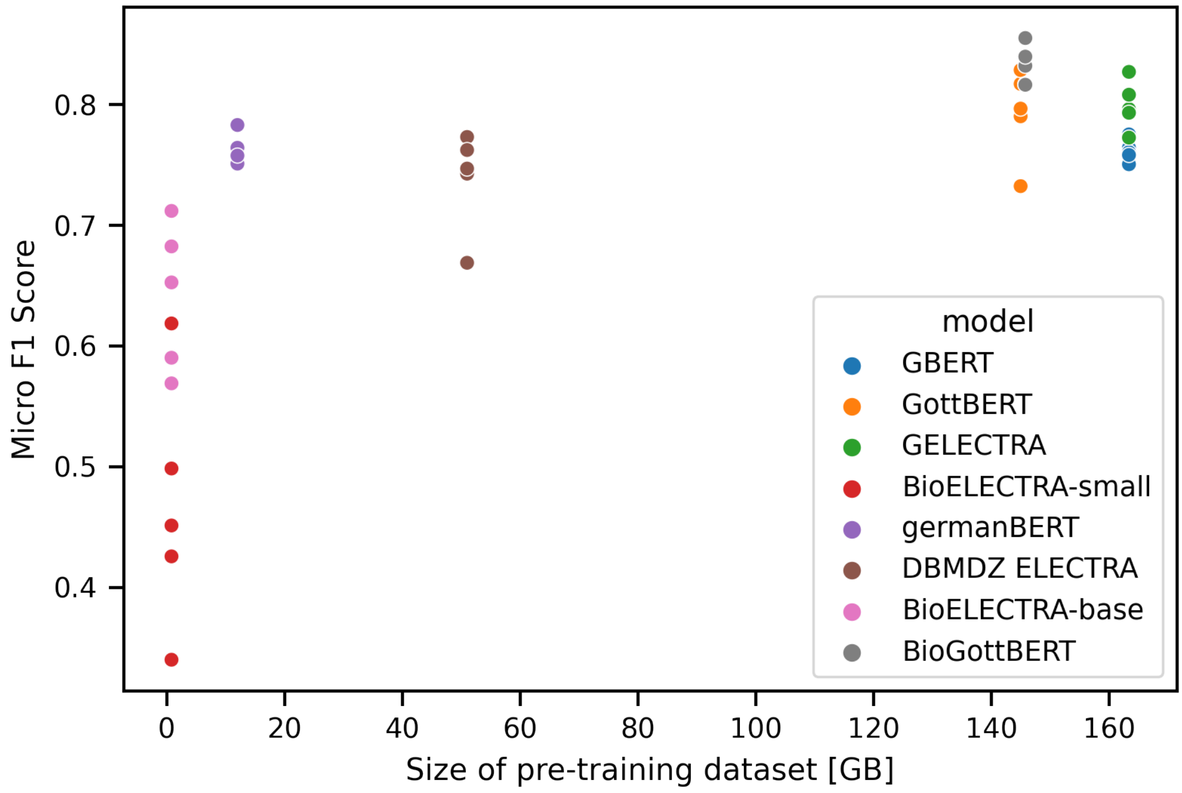


Supplementary Figure C.4: **Relationship between the size of the pre-training corpora and NER performance for BRONCO150.** Shown are the F1-scores for each entity with respect to the size of the pre-training corpora. Since nested cross-validation, we depict the results for each fold individually.

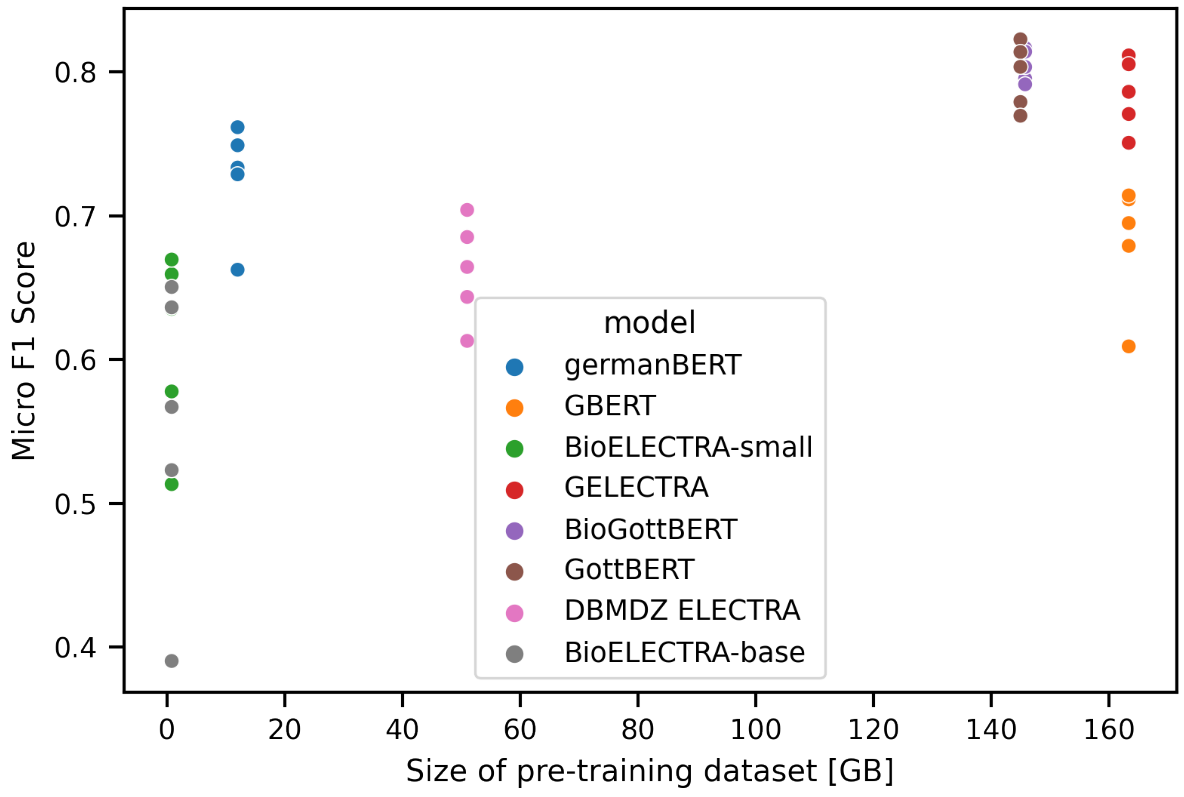


Supplementary Figure C.5: **Relationship between the size of the pre-training corpora and NER performance for ChaDL.** Shown are the F1-scores for each entity with respect to the size of the pre-training corpora. Since nested cross-validation, we depict the results for each fold individually.

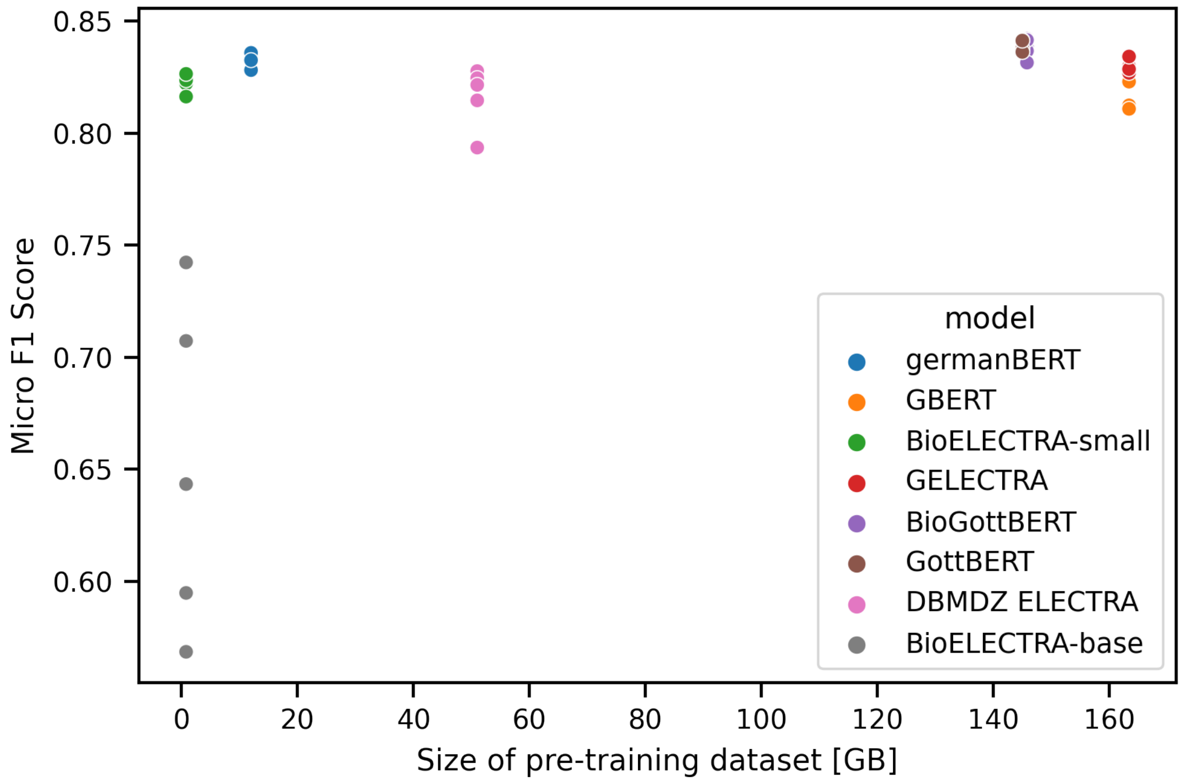


Supplementary Figure C.6: **Relationship between the size of the pre-training corpora and NER performance for GGPONC.** Shown are the F1-scores for each entity with respect to the size of the pre-training corpora. Since nested cross-validation, we depict the results for each fold individually.

# REFERENCES


[1] Marcin Junczys-Dowmunt et al. "Marian: Fast Neural Machine Translation in C++". In: *Proceedings of ACL 2018, System Demonstrations*. Melbourne, Australia, 2018. URL: https://arxiv.org/abs/1804.00344.

[2] Masoud Jalili Sabet et al. "SimAlign: High Quality Word Alignments without Parallel Training Data using Static and Contextualized Embeddings". In: *Proceedings of the 2020 Conference on Empirical Methods in Natural Language Processing: Findings*. Online: Association for Computational Linguistics, Nov. 2020, pp. 1627–1643. URL: https://www.aclweb.org/anthology/2020.findings-emnlp.147.

[3] Madeleine Kittner et al. "Annotation and initial evaluation of a large annotated German oncological corpus". In: *JAMIA Open* 4.2 (Apr. 2021). _eprint: https://academic.oup.com/jamiaopen/article-pdf/4/2/ooab025/38830128/ooab025.pdf. ISSN: 2574-2531. DOI: 10.1093/jamiaopen/ooab025. URL: https://doi.org/10.1093/jamiaopen/ooab025.

[4] Florian Borchert et al. "GGPONC: A Corpus of German Medical Text with Rich Metadata Based on Clinical Practice Guidelines". In: *arXiv:2007.06400 [cs]* (Nov. 2020). URL: http://arxiv.org/abs/2007.06400 (visited on 01/14/2021).

[5] Christina Lohr, Sven Buechel, and Udo Hahn. “Sharing Copies of Synthetic Clinical Corpora without Physical Distribution — A Case Study to Get Around IPRs and Privacy Constraints Featuring the German JSYNCC Corpus”. In: *Proceedings of the Eleventh International Conference on Language Resources and Evaluation (LREC 2018)*. LREC 2018. Miyazaki, Japan: European Language Resources Association (ELRA), May 2018. URL: https://aclanthology.org/L18-1201 (visited on 09/29/2021).

[6] James S Bergstra et al. “Algorithms for Hyper-Parameter Optimization”. In: (), p. 9.

[7] Loïc Lannelongue, Jason Grealey, and Michael Inouye. “Green Algorithms: Quantifying the Carbon Footprint of Computation”. In: *Advanced Science* 8.12 (2021), p. 2100707. ISSN: 2198-3844. DOI: 10.1002/advs.202100707. URL: https://onlinelibrary.wiley.com/doi/abs/10.1002/advs.202100707 (visited on 07/19/2022).
